# Supplementary material for: Content-rich biological network constructed by mining PubMed abstracts
Source: BMC Bioinformatics. 2004 Oct 8;5:147. doi: 10.1186/1471-2105-5-147 (PMC528731; doi:10.1186/1471-2105-5-147)
Supplement: Additional File 5 — The original Chilibot query results of the term "long-term potentiation (LTP)" and 22 other terms, limiting the latest references analyzed to the years 1990, 1995, 2000, and 2004. [file 1471-2105-5-147-S5.bz2 › chilibotAdditionalFile5/ltp2000/html/ERK_PKA.html]

 


 **ERK** and **PKA** 
  
Found 60 abstracts in PubMed,  **30 abstracts were retrieved and analyzed**.  


---

 Search Google  |
 PDF files only 
|  EDU domain only 

---

**Interactive relationship** (e.g. stimulation, inhibition, etc)

- In contrast, cAMP phosphorylated CREB via  **PKA**  directly but required a Rap1  **ERK**  pathway to activate a component downstream of CREB phosphorylation and CREB binding protein recruitment.  Ref: 10950954 J Biol Chem, 2000
- The results also demonstrated that in CRAC, MEK  **ERK**  pathway activated by TGFbeta is negatively regulated by  **PKA**  cascade but transactivated by PKC.  Ref: 10903877 Osteoarthritis Cartilage, 2000
- ... the increase in CREB phosphorylation was not, which suggests that in area CA3 the  **PKA**  cascade is not coupled to CREB phosphorylation via  **ERK** .  Ref: 10777769 J Neurosci, 2000
- However, incubation of slices with forskolin, an activator of the cAMP dependent protein kinase  **PKA**  cascade, did result in increases in active  **ERK**  and cAMP response element binding protein CREB phosphorylation in area CA3.  Ref: 10777769 J Neurosci, 2000
- Here we demonstrate that  **PKA**  is required for depolarization induced activation of both extracellular signal regulated kinase  **ERK**  and p38 mitogen activated protein MAP kinase in PC12 cells.  Ref: 10925166 Neurosci Lett, 2000
- Multiple signaling pathways have been shown to contribute to the regulation of CREB dependent transcription, including both protein kinase A  **PKA**  and mitogen activated protein MAP kinase extracellular signal regulated kinase  **ERK**  dependent kinase cascades.  Ref: 10950954 J Biol Chem, 2000
- Inhibition of protein kinase A  **PKA**  strongly diminished p38 activation mediated by DOR or ORL1 but had no significant effect on  **ERK**  activation, and protein kinase C PKC inhibitors potentiated stimulation of p38 while inhibiting activation of ERKs.  Ref: 10501195 J Neurochem, 1999
- In addition,  **ERK**  activation was also observed following treatment with phorbol dibutyrate PdBu and forskolin, activators of protein kinase C PKC and protein kinase A  **PKA** , respectively.  Ref: 10648887 Brain Res Mol Brain Res, 2000
- Activation of  **PKA**  by 8 bromo cyclic AMP or forskolin, and inhibition of PKC by calphostin C, resulted in a significant decrease in 3TP activity as well as in vitro  **ERK**  kinase activity in CRAC.  Ref: 10903877 Osteoarthritis Cartilage, 2000
- Activation of specific MEK  **ERK**  cascade is necessary for TGFbeta signaling and crosstalk with  **PKA**  and PKC pathways in cultured rat articular chondrocytes.  Ref: 10903877 Osteoarthritis Cartilage, 2000
- cAMP dependent protein kinase  **PKA**  inhibits T cell activation by phosphorylating ser 43 of raf 1 in the MAPK  **ERK**  pathway.  Ref: 11027949 Cell Signal, 2000
- This suppression appears to be a consequence of PGE2 elicited increases in  **PKA**  activity, which leads to inhibition of EGF induced activation of MAPK cascades at the level of Raf 1 and further affects downstream events including  **Erk**  2.  Ref: 10892858 Invest Ophthalmol Vis Sci, 2000

**Parallel relationship** (e.g. studied together, co-existance, homology, etc.)

- Calcium mediated phosphorylation of CREB through the  **PKA**  Rap1  **ERK**  pathway.  Ref: 10950954 J Biol Chem, 2000
- The kinases of this family that have been suggested to play a role in apoptosis are the mitogen activated protein kinase MAPK family, specifically p42 44  **ERK** , p38 MAPK and c Jun N terminal kinase JNK, cyclic AMP dependent protein kinase  **PKA** , protein kinase B PKB, or Akt and protein kinase C PKC.  Ref: 10739649 Exp Cell Res, 2000
- Phosphorylation consensus sites for various kinases exist within the sequence of the potassium channel subunit Kv4.2, including consensus sites for extracellular signal regulated kinase mitogen activated protein kinase  **ERK**  MAPK, protein kinase A  **PKA** , protein kinase C PKC, and calcium calmodulin dependent kinase II CaMKII, and kinase assays have shown that particular amino acids of the consensus sites are bonafide phosphorylation sites in vitro.  Ref: 11040264 Learn MemLearn Mem, 2000
- Isoproterenol s effects on  **PKA** , PKC and  **ERK**  dependent activities were blocked by propranolol, a betaAR antagonist.  Ref: 10407186 Brain Res Mol Brain Res, 1999
- As with the dependence of  **Erk**  2 activity on  **PKA**  activity, in the presence of H 89, the EGF induced Raf 1 activation was significantly enhanced.  Ref: 10892858 Invest Ophthalmol Vis Sci, 2000
- Thus, we further show that, in both PC12 cells and hippocampal neurons, depolarization induced calcium influx stimulates  **ERK**  activity in a  **PKA**  dependent manner.  Ref: 10652372 J Biol Chem, 2000
- For example, the stratum lacunosum moleculare, which receives inputs from the entorhinal cortex via the perforant pathway, displays relatively little  **ERK**  phosphorylated Kv4.2 or  **PKA**  carboxy terminal phosphorylated Kv4.2.  Ref: 11040264 Learn MemLearn Mem, 2000
- In contrast to the depolarization induced signaling, the  **ERK**  and PYK2 activation by bradykinin 1 microM, a G protein coupled receptor agonist, was not blocked by  **PKA**  inhibition.  Ref: 10925166 Neurosci Lett, 2000
- ... the blockade of PKC,  **PKA** , and  **ERK**  had little effect.  Ref: 11006016 Cell Immunol, 2000
- We have developed antibodies recognizing Kv4.2 triply phosphorylated at the three  **ERK**  sites as well as two antibodies recognizing singly phosphorylated Kv4.2 channels at the  **PKA**  sites one amino terminal and one carboxy terminal.  Ref: 11040264 Learn MemLearn Mem, 2000
- We conclude that cAMP agonists stimulate the proliferation of ADPKD but not HKC epithelial cells through  **PKA**  activation of the  **ERK**  pathway at a locus distal to receptor tyrosine kinase.  Ref: 10760082 Kidney Int, 2000
- We conclude that ISO, regulates the Na, K ATPase in AEC via  **PKA** ,  **ERK**  and rapamycin sensitive mechanisms.  Ref: 11119725 FEBS Lett, 2000
- To examine the possibility that whether activation of Raf 1 and  **ERK**  1, ERK2 and p38 MAPK can be regulated directly or and differently by PKC and  **PKA**  pathways, herbimycin A, a potent PTK inhibitor at 2 mumol L concentration was utilized to block Ras Raf 1 MAPK cascade.  Ref: 10684111 Shi Yan Sheng Wu Xue Bao, 1997
- The results in this paper indicate that LPS induced NF kappa B binding activity was independent of PKC,  **PKA** ,  **ERK** , and p38 MAPK, but was regulated by proteasome.  Ref: 11006016 Cell Immunol, 2000
- Therefore, CPO activates the  **ERK**  44 42 signaling cascade in CHOK1 cells via a pathway dependent on P13 K, PKC, and MEK but not requiring  **PKA**  or the human M2 muscarinic receptor.  Ref: 11083088 J Biochem Mol Toxicol, 2000
- On the other hand, either inhibition of PGE2 production with AACOCF3 or H 89, a  **PKA**  inhibitor, enhanced EGF induced  **Erk**  2 activity.  Ref: 10892858 Invest Ophthalmol Vis Sci, 2000
- Similarly, of the three antibodies tested, the soma of CA3 neurons are primarily recognized by the  **ERK**  triply phosphorylated Kv4.2 antibody, and the mossy fiber inputs to CA3 are primarily recognized by the carboxy terminal  **PKA**  phosphorylated Kv4.2.  Ref: 11040264 Learn MemLearn Mem, 2000
- The effects of exogenous PGE2, inhibition of PGE2 synthesis, and modulation of protein kinase A  **PKA**  activity on EGF induced  **Erk**  2 activation were assessed by immunoprecipitation, kinase assays, and Western blot analysis.  Ref: 10892858 Invest Ophthalmol Vis Sci, 2000
- An intricate crosstalk between  **PKA**  and the mitogen activated protein kinase MAPK  **ERK**  pathway, the p70 S6 kinase pathway and cyclin dependent kinases has been described.  Ref: 10405757 Cell Signal, 1999
- ISO increased  **ERK**  activity in AEC by 10 min via a beta adrenergic receptor, protein kinase A  **PKA**  dependent mechanism.  Ref: 11119725 FEBS Lett, 2000
- In addition, the ability of procaterol to dephosphorylate  **ERK**  in an RP 8 Br cAMPS and PTX insensitive manner suggests that this may contribute to the anti spasmogenic activity of beta adrenoceptor agonists by activating a novel  **PKA**  independent pathway.  Ref: 11053220 Br J Pharmacol, 2000
- Whereas a prolonged elevation of cAMP in VSMC resulted in a protein kinase A  **PKA**  dependent induction of expression of two PDE4D variants PDE4D1 and PDE4D2, simultaneous activation of both the cAMP  **PKA**  and PKC Raf MEK  **ERK**  signaling cascades blunted this cAMP mediated increase in PDE4D expression.  Ref: 10851231 J Biol Chem, 2000
